# A test of a conceptual model of uncertainty, benefit and burden in children and young people with juvenile dermatomyositis

**DOI:** 10.1093/rap/rkag070

**Published:** 2026-06-10

**Authors:** Polly Livermore, Klaudia H Kupiec, Kathleen Mulligan, Shashi P Hirani, Faith Gibson, Claire Deakin, Deborah Ridout, Andrea M Knight, Lucy R Wedderburn, Brian M Feldman, the Juvenile Dermatomyositis Cohort Biomarker Study and Repository (JDCBS), Kate Armon, Louise Coke, Julie Cook, Amy Nichols, Vanja Briggs, Emily Tropman, Liza McCann, Ian Roberts, Eileen Baildam, Louise Hanna, Olivia Lloyd, Susan Wadeson, Michelle Andrews, Olivia Lloyd, Jane Roach, Beverley Almeida, Phil Riley, Ann McGovern, Verna Cuthbert, Precious Iheke, Clive Ryder, Janis Scott, Beverley Thomas, Professor Taunton Southwood, Eslam Al-Abadi, Ruth Howman, Sue Wyatt, Gillian Jackson, Mark Wood, Tania Amin, Vanessa VanRooyen, Deborah Burton, Louise Turner, Heather Rostron, Sarah Hanson, Joyce Davidson, Janet Gardner-Medwin, Neil Martin, Sue Ferguson, Liz Waxman, Michael Browne, Roisin Boyle, Emily Blyth, Susanne Cathcart, Kirsty McLellan, Jaclyn Keightley, Mark Friswell, Professor Helen Foster, Alison Swift, Sharmila Jandial, Vicky Stevenson, Debbie Wade, Ethan Sen, Eve Smith, Lisa Qiao, Stuart Watson, Claire Duong, Stephen Crulley, Andrew Davies, Miss Caroline Miller, Lynne Bell, Flora McErlane, Sunil Sampath, Josh Bennet, Sharon King, Christopher Long, Lesley Brindley, Helen Venning, Rangaraj Satyapal, Elizabeth Stretton, Mary Jordan, Ellen Mosley, Anna Frost, Lindsay Crate, Kishore Warrier, Stefanie Stafford, Brogan Wrest, Chia-Ping Chou, Paul Pryce, Professor Lucy Wedderburn, Clarissa Pilkington, Nathan Hasson, Muthana Al-Obadi, Giulia Varnier, Sandrine Lacassagne, Sue Maillard, Lauren Stone, Elizabeth Halkon, Virginia Brown, Audrey Juggins, Sally Smith, Sian Lunt, Elli Enayat, Hemlata Varsani, Laura Kassoumeri, Miss Laura Beard, Katie Arnold, Yvonne Glackin, Stephanie Simou, Beverley Almeida, Kiran Nistala, Raquel Marques, Claire Deakin, Parichat Khaosut, Stefanie Dowle, Charalampia Papadopoulou, Shireena Yasin, Christina Boros, Meredyth Wilkinson, Chris Piper, Cerise Johnson-Moore, Lucy Marshall, Kathryn O’Brien, Emily Robinson, Dominic Igbelina, Polly Livermore, Socrates Varakliotis, Rosie Hamilton, Lucy Nguyen, Dario Cancemi, Ovgu Kul Cinar, Elena Moraitis, Serena Cruickshank-Hull, Klaudia Kupiec, Hannah Peckham, Qiong Wu, Melissa Kartawinata, Bethany Jebson, Nia Evans, Chadwick Pils, Persephone Jenskins, Afroditi Barmpakou, Ali Mulla Issa, Senne Cuyx, Ryan Lethem, Nagesha Muniyappa, Zahoor Khan, Mashal Shamsuddin, Chenqu Suo, Mariyah Albrahim, Nkechi Maduaka, Lilase Maduaka, Samantha Cooray, Rebecca Dancey, Kevin Murray, Coziana Ciurtin, John Ioannou, Caitlin Clifford, Linda Suffield, Maryam Butt, Sydnee Pope, Laura Hennelly, Helen Lee, Sam Leach, Helen Smith, Anne-Marie McMahon, Heather Chisem, Jeanette Hall, Amy Huffenberger, Nick Wilkinson, Emma Inness, Eunice Kendall, David Mayers, Ruth Etherton, Danielle Miller, Kathryn Bailey, Jacqui Clinch, Natalie Fineman, Helen Pluess-Hall, Suzanne Sketchley, Melanie Marsh, Anna Fry, Maisy Dawkins-Lloyd, Mashal Asif, Gigi Leung, Alexander Smith, Joyce Davidson, Margaret Connon, Lindsay Vallance, Kirsty Haslam, Charlene Bass-Woodcock, Trudy Booth, Louise Akeroyd, Alice Leahy, Amy Collier, Rebecca Cutts, Emma Macleod, Hans De Graaf, Brian Davidson, Sarah Hartfree, Elizabeth Fofana, Lorena Caruana, Catriona Anderson, Jayne MacMahon, Peter Bale

**Affiliations:** Infection, Immunity and Inflammation Research and Teaching Department, UCL Great Ormond Street Institute of Child Health, London, UK; Paediatric Rheumatology, Great Ormond Street Hospital NHS Foundation Trust, London, UK; Centre for Outcomes and Experience Research in Children’s Health, Illness and Disability, Great Ormond Street Hospital for Children NHS Foundation Trust, London, UK; National Institute for Health Research Biomedical Research Centre at Great Ormond Street Hospital, London, UK; Infection, Immunity and Inflammation Research and Teaching Department, UCL Great Ormond Street Institute of Child Health, London, UK; Paediatric Rheumatology, Great Ormond Street Hospital NHS Foundation Trust, London, UK; Centre for Outcomes and Experience Research in Children’s Health, Illness and Disability, Great Ormond Street Hospital for Children NHS Foundation Trust, London, UK; National Institute for Health Research Biomedical Research Centre at Great Ormond Street Hospital, London, UK; School of Health and Medical Sciences, Department of Health Services Research and Management, City St George’s, University of London, London, UK; School of Health & Medical Sciences, East London NHS Foundation Trust, London, UK; Department of Global, Public and Population Health and Policy, School of Health and Medical Sciences, City St George’s, University of London, London, UK; Centre for Outcomes and Experience Research in Children’s Health, Illness and Disability, Great Ormond Street Hospital for Children NHS Foundation Trust, London, UK; School of Health Sciences, University of Surrey, Guildford, Surrey, UK; Infection, Immunity and Inflammation Research and Teaching Department, UCL Great Ormond Street Institute of Child Health, London, UK; National Institute for Health Research Biomedical Research Centre at Great Ormond Street Hospital, London, UK; Centre for Adolescent Rheumatology at UCL, UCL Hospital and Great Ormond Street Hospital, London, UK; School of Population Health, Faculty of Medicine and Health, University of New South Wales, Sydney, New South Wales, Australia; Population, Policy & Practice Department, UCL Great Ormond Street Institute of Child Health, London, UK; The Hospital for Sick Children, Department of Paediatrics, University of Toronto, Toronto, Ontario, Canada; Infection, Immunity and Inflammation Research and Teaching Department, UCL Great Ormond Street Institute of Child Health, London, UK; National Institute for Health Research Biomedical Research Centre at Great Ormond Street Hospital, London, UK; Centre for Adolescent Rheumatology at UCL, UCL Hospital and Great Ormond Street Hospital, London, UK; The Hospital for Sick Children, Department of Paediatrics, University of Toronto, Toronto, Ontario, Canada

**Keywords:** uncertainty, illness burden, quality of life, depression, anxiety, paediatric rheumatology, childhood chronic conditions

## Abstract

**Objectives:**

Mental health concerns are highly prevalent in children and young people (CYP) with chronic conditions. This is further exacerbated by perceived feelings of illness uncertainty and illness burden, which have negative implications for CYP with childhood-onset chronic conditions and their families. Understanding the mechanisms that reduce health-related quality of life (HRQoL) and identifying resilience factors is important to improve outcomes for these vulnerable populations. The current study aimed to assess HRQoL in CYP with juvenile dermatomyositis (JDM) and to test a new conceptual model of illness representations and resilience in depression and anxiety.

**Methods:**

CYP ages ≥8 years who were enrolled in the UK-wide JDM cohort and Biomarker Study (JDCBS) in 15 UK National Health Service tertiary paediatric rheumatology services were invited to complete five validated paediatric measures: PedsQL 4.0 Core, PedsQL 3.0 Rheumatology Module, Paediatric Index of Emotional Distress (PI-ED), Childhood Uncertainty in Illness Scale (CUIS) and Benefit and Burden Scale for Children (BBSC). Data were analysed using descriptive statistics and structural equation modelling.

**Results:**

Results showed that the HRQoL in CYP with JDM is affected as a result of their chronic condition. Moreover, the proposed model revealed that illness uncertainty and burden are key driving factors that cause an increase in anxiety and depression, while benefit finding did not ameliorate poor mental health outcomes.

**Conclusion:**

Addressing illness uncertainty and reducing the perceived disease burden is vital to improve HRQoL and well-being in CYP with JDM who struggle to cope with the unpredictable nature of their disease.

Key messages

- Illness uncertainty and burden increase anxiety and depression in children and young people (CYP) with JDM.
- Benefit finding does not improve health-related quality of life or well-being in CYP with JDM.
- Interventions need to focus on addressing mental health alongside physical health to improve outcomes.

## Introduction

Advancements in healthcare have improved outcomes for children with chronic conditions by increasing survival rates. However, the growing prevalence of childhood chronic diseases is presenting a global burden [1, 2]. Chronic conditions are long-term conditions that often require ongoing management and treatment. Lack of proper management of childhood chronic conditions is associated with decreased health-related quality of life (HRQoL), impairing a child’s physical health as well as developmental, social and mental well-being [3]. Moreover, these conditions impact upon the whole family and are associated with economic challenges. Childhood chronic conditions include conditions such as cancer, congenital heart diseases, cystic fibrosis, diabetes, asthma and rheumatological conditions. Rheumatological conditions are autoimmune and autoinflammatory conditions and include common conditions such as childhood-onset arthritis, also known as juvenile idiopathic arthritis (JIA), as well as rarer conditions such as juvenile idiopathic inflammatory myopathies (JIIMs) [3]. JIIMs such as juvenile dermatomyositis (JDM), characterised by muscle and skin inflammation, can cause psychological distress, with significant mental health symptoms such as irritability and low mood [4, 5]. Recent literature suggests that high proportions of those with JIIMs report psychological distress at levels that warrant mental health referral [6], therefore understanding predictors for impairment of HRQoL is important.

Despite healthcare professionals rating timely diagnosis as the most important aspect of high-quality care, adult family members of patients with juvenile inflammatory myositis believe that HRQoL is the most important aspect of healthcare [7]. This is relevant as children and young people (CYP) with JDM have been found to have a significantly lower HRQoL, with poorer physical and psychological well-being, than healthy controls [8–10]. However, these prior studies assessed HRQoL using proxy measures completed by adult caregivers rather than addressing specifically the views of children, young people or young adults living with JDM. The literature highlights that parents generally underestimate the impact of chronic disease on HRQoL compared with their children. In a study by Waters *et al.* [11], CYP were found to be much less optimistic about their health and well-being than their parents and more likely to be sensitive to pain, mental health problems and the impact of their health on family activities. Proxy reports can also be confounded by the parents’ psychological state, with depression being found to negatively correlate with parent proxy health reports [12]. A study by Kountz-Edwards *et al.* [9] found that having a child with active rheumatological disease can negatively affect a parent’s mood.

As it is also known that CYP may not always feel comfortable sharing their own difficulties or mental health needs with their parents [13], it is important to fully understand their perspectives. Understanding poor mental health outcomes in CYP with chronic conditions is important, as 62% of these are more likely to be diagnosed with a mental health disorder [14]. Research investigating mental health prevalence rates in CYP with JDM and other paediatric rheumatological conditions (JIA and SLE) found that 35% of CYP are diagnosed with depression as assessed by a clinician, with an additional 18% reporting self-diagnosed symptoms, while 39% of this sample had clinically diagnosed anxiety and another 27% reported clinically relevant anxious symptoms [13]. The current study has built upon previous work that explored the lived experience of a small group of CYP with JDM through in-depth interviews in one tertiary centre in the UK [15]. Livermore *et al.*’s [15] qualitative appraisal of interview data with CYP described living with the disease as a rollercoaster journey of ups and downs and identified five main themes: confusion, feeling different, being nauseous, anxiety related to medications and uncertainty.

The relationship of self-perceived illness uncertainty and its impact on mental health have been investigated in CYP with chronic conditions such as cancer and asthma, but little is known about the relationship between these concepts in rheumatological conditions such as JDM. For example, a study testing a conceptual model of uncertainty in CYP with cancer showed that uncertainty is associated with increased levels of emotional distress [16]. Research in CYP with asthma and type 1 diabetes suggests that uncertainty is associated with higher levels of depression [17–19], anxiety [17, 20] and psychological distress [21, 22]. Considering the unpredictability of the disease course for CYP with JDM, the current study aims to investigate levels of self-perceived uncertainty in children. In paediatric rheumatology, a study by Fedele *et al.* [23] found that caregiver uncertainty has an impact on parent’s and children’s levels of distress, while parent distress has also been shown to be associated with depressive symptoms in children with rheumatological conditions and high levels of child-perceived uncertainty [24]. Similarly, disease burden is often addressed from the perspective of parents and caregivers instead of assessing the impact of the disease burden on CYP themselves. For example, a recent study found that caregiver burden and caregiver uncertainty impacted upon the capacity of caregivers of children with chronic uveitis [25]. Research with adult populations with cancer found that self-perceived burden and illness uncertainty was negatively associated with HRQoL [26].

In order to develop suitable interventions to support mental health in CYP with chronic conditions, it is also vital to understand mechanisms that can improve outcomes. Research focusing on resilience and adaptive coping suggests that benefit finding can be a useful measure to assess resilience. Benefit finding refers to a way of coping referring to a positive outlook in relation to life changes from adversity and conceptualised as personal growth from adversity. Benefit finding has been found to ameliorate symptoms of anxiety and depression in adult cancer populations [27]. Preliminary findings investigating benefit finding in children with cancer found a negative association between benefit finding and anxiety [28].

**Figure 1:**
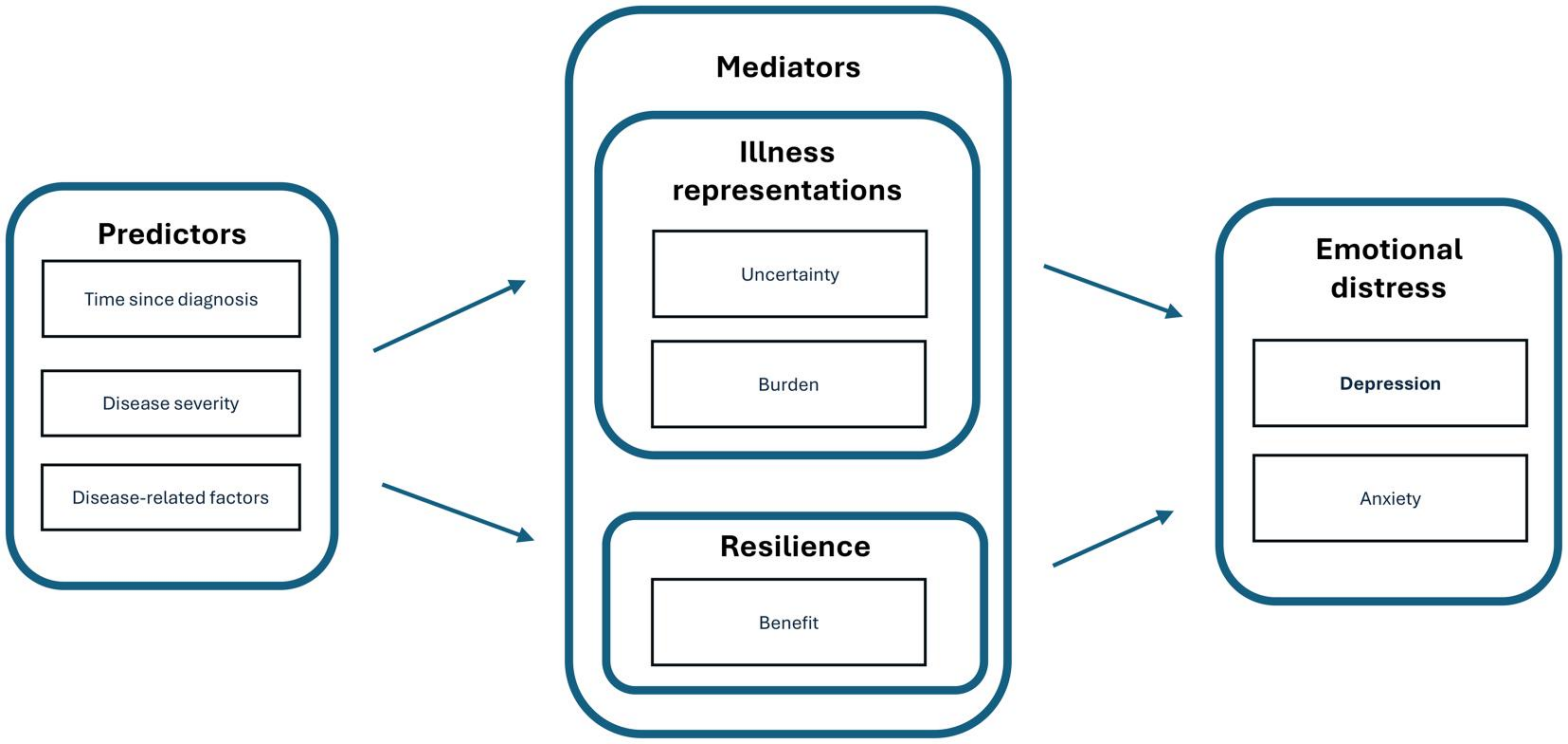
A conceptual model of illness representations and resilience in CYP with JDM

The objective of this current work was to investigate resonance with the themes found in the earlier study [15], specifically the impact upon HRQoL and perceptions of uncertainty, burden and benefit finding in a UK-based sample of CYP with JDM. Based on previous research, a new model was proposed (see Fig. 1). The proposed model was adapted from the conceptual models from Stewart *et al.* [16], which was originally derived from Mishel’s (1988) [29] uncertainty in illness theory, and Zhu *et al.*’s [30] model on benefit finding and mental health. The current model investigated health-related determinates related to illness representations as a predictor of emotional distress in CYP with JDM while also exploring the role of self-perceived benefit as a mediating factor. The specific aims of the study were 2-fold: to describe the HRQoL of CYP with JDM and to test a new model of uncertainty, benefit and burden in relation to mental health outcomes of CYP with JDM.

## Methods

### Setting

The Juvenile Dermatomyositis Cohort and Biomarker Study () is a national cohort study and sample repository, established in 2000 in the UK. This is an inception cohort study of children diagnosed with idiopathic inflammatory myopathies, collecting clinical and biological data longitudinally, with paired samples from diagnosis, and serially over time [31, 32]. This is a cross-sectional study nested within JDCBS. At the time, there were 628 enrolled and consented patients, >6000 recorded clinic visits, 2000 blood samples and 150 muscle biopsies from 15 UK centres.

### Participants

Inclusion criteria for participants with idiopathic inflammatory myopathies were all CYP >8–<19 years of age from the UK, who were enrolled in the JDCBS and had previously given consent to be approached for research.

### Recruitment

The database was searched by the research lead (P.L.) and a list of all eligible CYP compiled. Initial contact was made by a study information postcard alerting families to the aim of the study and providing an opt-out email if families did not want to participate. Families who did not opt out were sent a postal pack of questionnaires containing an information sheet, all questionnaires, a return prepaid envelope and a link to online electronic versions via an approved university survey software (Opinio). In addition, questionnaire packs were also disseminated via rheumatology nurse specialists in local hospitals.

### Ethics and consent

A substantial amendment to the JDCBS original ethical approval (North Yorkshire REC, ref 1/3/22) was obtained, along with approval from the JDCBS Steering Group, University Ethical Approval and the sponsor Trust Research & Development Department. Participants and their parents had previously given consent or assent (if <16 years of age), including parental or self-consent to be contacted for future studies, and accompanying patient information reminding both the CYPs and their parents/caregivers that completion of these measures was voluntary.

### Description of study measures

Questionnaire packs consisted of five validated measures all previously used with child populations:

- Pediatric Quality of Life Inventory (PedsQL) 4.0 Generic Core Scale (PedsQL, ©1998 J. W. Varni, PhD. All rights reserved). There are two age-appropriate surveys (8–12 years and 13–18 years), with 23 ordinal questions in total, which make up four domains (physical functioning, emotional functioning, social functioning, school functioning) and two composite scores (physical and psychosocial). Scores are reverse scored and linearly transformed to a 0–100 scale, with higher scores indicating better HRQoL [33, 34].
- PedsQL 3.0 Rheumatology Module. Similar to the PedsQL, there are two age-appropriate surveys (8–12 years and 13–18 years), with 22 ordinal questions and five domains (pain, daily activities, treatment, worry and communication). Scores are again reverse scored and linearly transformed to a 0–100 scale, with higher scores indicating better rheumatology HRQoL [35].
- Paediatric Index of Emotional Distress (PI-ED). This is a self-report measure for CYP ≥8 years of age, with 14 ordinal questions. Summing the total score provides a result for overall emotional distress of 0–42, with higher scores indicating greater emotional distress. The PI-ED is based on the Hospital Anxiety and Depression Scale, which was created, tested and validated for adult populations attending routine medical appointments [36]. The PI-ED also consists of subscales (seven questions each) screening for anxious and depressive symptoms [37].
- Childhood Uncertainty in Illness Scale (CUIS). This is a self-reported, 23-item ordinal tool for children ages 8–17 years, used to address four components of children’s perceived uncertainty regarding their illness: ambiguity, lack of clarity, lack of information and unpredictability. Participants rate items on a 1–5 scale with a total score being calculated at the end. Higher scores indicate greater levels of uncertainty [21, 38, 39].
- Benefit and Burden Scale for Children (BBSC). The BBSC is a 20-item self-report instrument originally developed for a cancer study in children ages 8–18 years, with 20 items: 10 each for benefit finding—measuring how well children adapt—and illness burden, assessed on a 5-point ordinal scale. A total sum of the scores provides separate benefit and burden scores between 10 and 50; the higher the scores, the higher the perceived benefit and burden [40, 41].

As clinical data could be accessed via the JDCBS, the decision was made to not collect any current objective measures of JDM activity to avoid overburdening participants. Data extracted from the JDCBS included sex, date of onset of disease, date of diagnosis and methotrexate therapy at any point (yes or no). Initial baseline visit details included date, Childhood Myositis Assessment Scale (CMAS; 0–52, lower scores indicate worse disease activity), physician visual analogue scale (VAS) (0–100, higher scores indicate worse disease activity), patient/parent VAS score (0–100, higher scores indicate worse disease activity) and rash present (yes or no). The same data were extracted from the last recorded visit relative to data from self-reported questionnaires.

### Statistical analysis

JASP version 0.95.4.0, a freely available statistical software package (https://jasp-stats.org/), was used to run all analyses, including structural equation modelling (SEM) [42]. SEM was used to test the proposed conceptual model (Fig. 1) to determine its fit. SEM is used to test statistical frameworks designed to simultaneously test and estimate both direct and indirect (mediated) relationships among a set of variables [43]. The approach accommodates relationships between observed measures as well as latent constructs—unobserved variables inferred from a set of empirical indicators—thereby enabling the representation of theoretically motivated measurement structures alongside substantive path models. In SEM, the researcher specifies an evidence-based hypothesised model, commonly expressed as a path diagram, from which model parameters are estimated and overall model fit is evaluated against the observed data. As there is no single test that measures and evaluates the model fit, a range of different fit indices including the chi-squared (χ^2^) statistic and degrees of freedom (df), goodness-of-fit index (GFI), root mean squared error of approximation (RMSEA) and standardized root mean square residual (SRMR) were used to determine absolute fit indices [43]. Additionally, the comparative normed fit index (CFI) and the Tucker–Lewis index (TLI) as incremental fit indices were assessed [43]. Table 1 shows cut-offs and recommendations of fit indices as evaluated by Schermelleh-Engel *et al.* [43].

**Table 1:** Adapted goodness-of-fit measures and recommendations from Schermelleh-Engel *et al.* [43] (published under a CC BY-SA licence).

Fit measure	Good fit	Acceptable fit
χ^2^	≥0–≤2 df	>2 df–≤3 df
χ^2^/df	>0.05–≤2	>2–≤3
GFI	≥0.95–≤1.00	≥0.90–<0.90
RMSEA	≥0–≤0.05	>0.05–≤0.08
CFI	≥0.97–≤1.00	≥0.95–<0.97
TLI	≥0.95–1.00	≥0.90–<0.95
SRMR	≥0–≤0.05	>0.05–≤0.10

## Results

**Figure 2:**
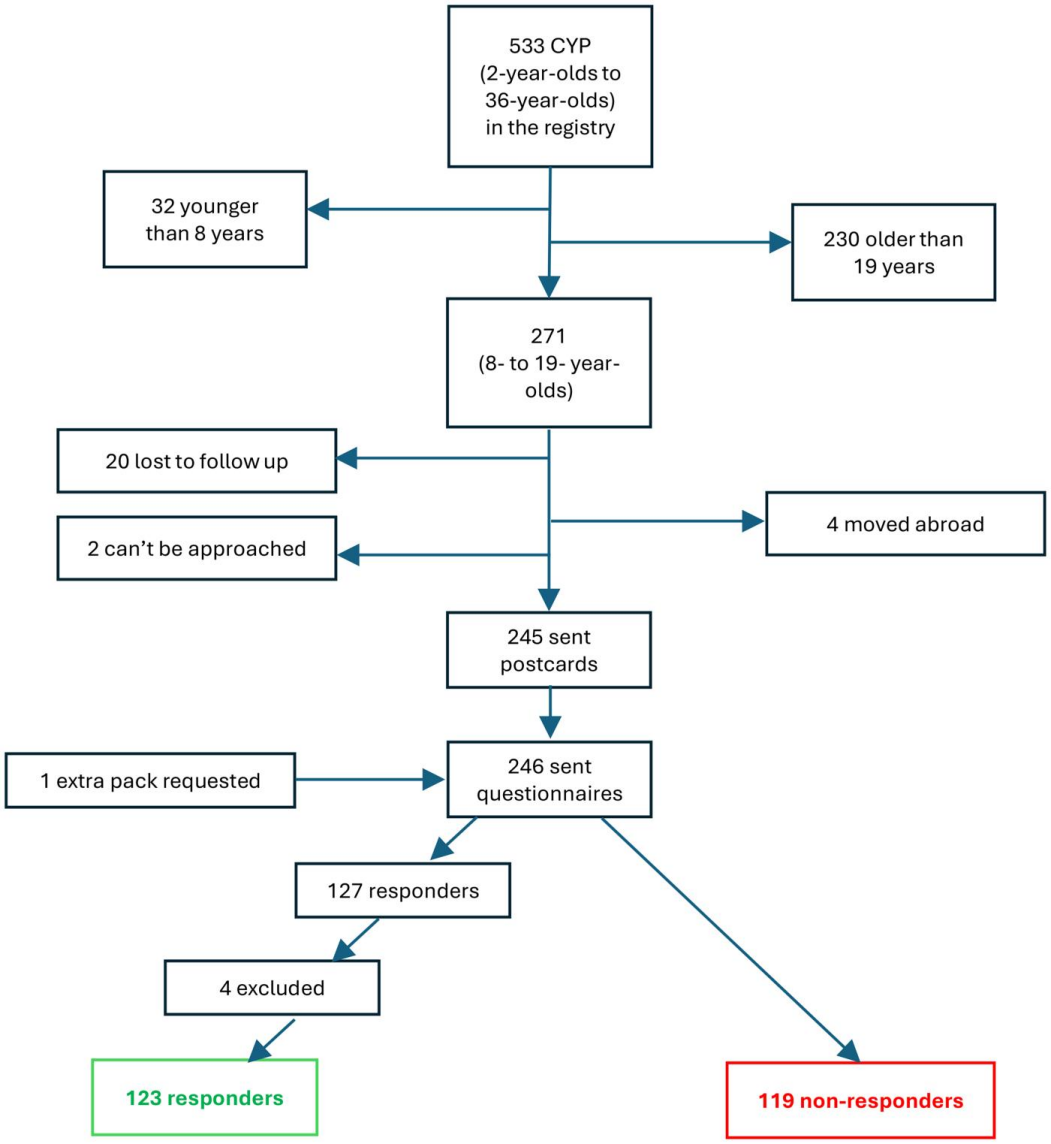
Recruitment flow chart

No e-mails were received from parents of CYP requesting to opt out from any of the 15 centres, therefore questionnaire packs were sent to the 245 potentially eligible participants (of the 533 in the registry at this time, with an age range of 2–26 years). Postal packs were addressed to the known parent at that address, who was asked to give it to their child. Questionnaires were returned from 126 individuals; however, 3 were excluded (1 only had demographic data completed, 1 was unreadable and 1 was from a patient later diagnosed as having SLE). This left a sample of 123 responders (50.8%) and 119 non-responders. The response rate across centres was 48–83%. Demographic data of respondents/non-respondents, reported in Table 2, showed minimal differences between the two groups except that responders had a more recent clinic visit than non-responders. A flow chart of recruitment can be found in Fig. 2.

**Table:** **Table 2** Demographic and clinical characteristics of responders and non-responders.

Characteristics	Non-responder (*n* = 119)	Responder (*n* = 123)
Female, *n* (%)	74 (62)	82 (67)
Male, *n* (%)	45 (38)	41 (33)
Received methotrexate at any point, *n* (%)	109/117 (93)	115/119 (97)
Age when questionnaire completed, years, mean (s.d.)	13.36 (3.38)	13.42 (3.32)
Age at diagnosis, years, mean (s.d.)	7.06 (3.38)	7.59 (3.73)
Time since diagnosis, years, mean (s.d.)	7.17 (3.70)	6.58 (3.93)
Time since last clinic visit, years, mean (s.d.)	1.80 (2.54)*	1.11 (1.73)*
CMAS at baseline, mean (s.d.)	32.61 (16.88)	32.09 (15.51)
Physician VAS at baseline, mean (s.d.)	3.78 (2.80)	3.67 (2.88)
Patient/parent VAS at baseline, mean (s.d.)	3.42 (2.93)	2.99 (2.96)

* *P* < 0.05.

**Figure 3:**
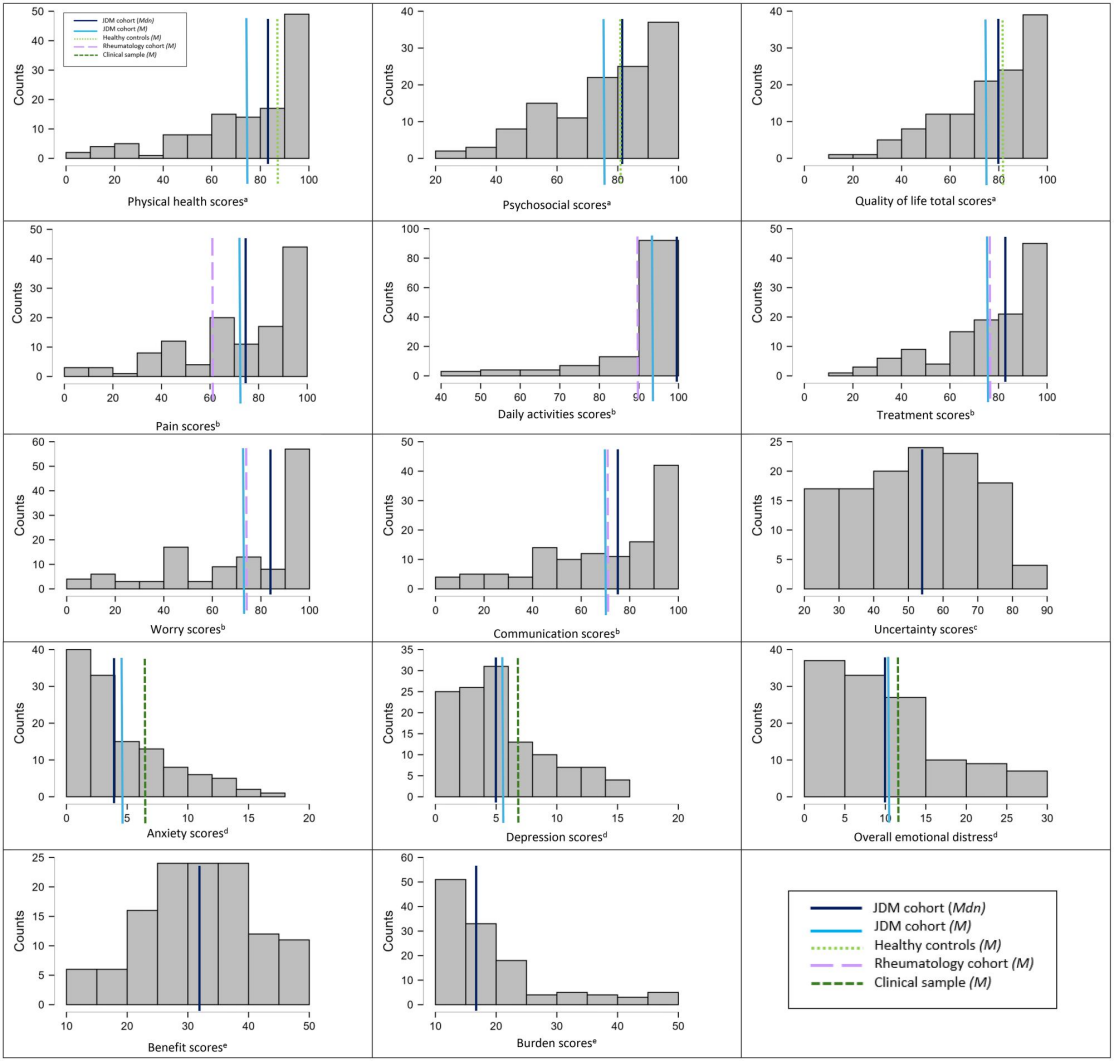
Histograms show scores across self-reported outcomes. Dark blue lines show the overall median score for each outcome measure. Mean values are shown where a comparison was possible between the current JDM cohort against a healthy, age-matched cohort [44], a comparative sample of CYP with a range of rheumatological conditions [35] or a clinical sample that was recruited from paediatric outpatient departments and psychology services [37]. Mean scores from the current JDM cohort are represented by light blue lines, healthy controls are represented by light green, the comparative rheumatology sample is represented by purple and the clinical sample by dark green. Self-report data from CYP themselves (instead of parent-proxy data) has been used where available. ^a^Subscores and total score as measured by the PedsQL 4.0 Generic Core Scale. ^b^Subscores as measured by the PedsQL 3.0 Rheumatology Module. ^c^Uncertainty as measured by the CUIS. ^d^Subscores and main score as measured by the PI-ED. ^e^Subscores as measured by the BBSC.

All returned questionnaires were scored. Descriptive analyses showing outcomes from the questionnaire measures can be seen in Fig. 3. Comparisons, where possible, were made between the current JDM cohort *vs* either a healthy, age-matched cohort [44], a comparative sample of CYP with a range of rheumatological conditions [35] or a clinical sample that was recruited from paediatric outpatient departments and psychology services [37]. The comparative cohorts were chosen based on criteria evaluating the selected questionnaires in relation to consistency, reliability and validity from large-scale cohorts. The cohorts varied between comparisons due to their availability and reliability testing. A UK-wide sample has been used to validate the psychometric properties of the UK version of the PedsQL Generic Core Scale, which was administered to 1399 children and 970 of their parents and included healthy controls [44]. However, the PedsQL 3.0 Rheumatology has not been validated with healthy controls, as the measure was designed to specifically address paediatric rheumatology–related HRQoL, therefore a different cohort was chosen. Varni *et al.* [35] validated the PedsQL 3.0 Rheumatology by administering the questionnaire to 231 children and 244 parents who were recruited from paediatric rheumatology clinics and their dataset has been used as a comparative sample here. The PI-ED was adapted for paediatric cohorts and evaluated with both healthy school-age children (*n *= 1026) as well as clinical samples (*n *= 143) [37], which is why it has been chosen as a comparative cohort in the current study.

### SEM

The predicted model (Fig. 1) relied on five latent variables (disease severity, disease-related factors, illness representations, resilience and emotional distress) with different predicted paths. Emotional distress as indicated by the anxiety and depressive symptom subscales was the primary (latent) outcome in the model.

**Figure 4:**
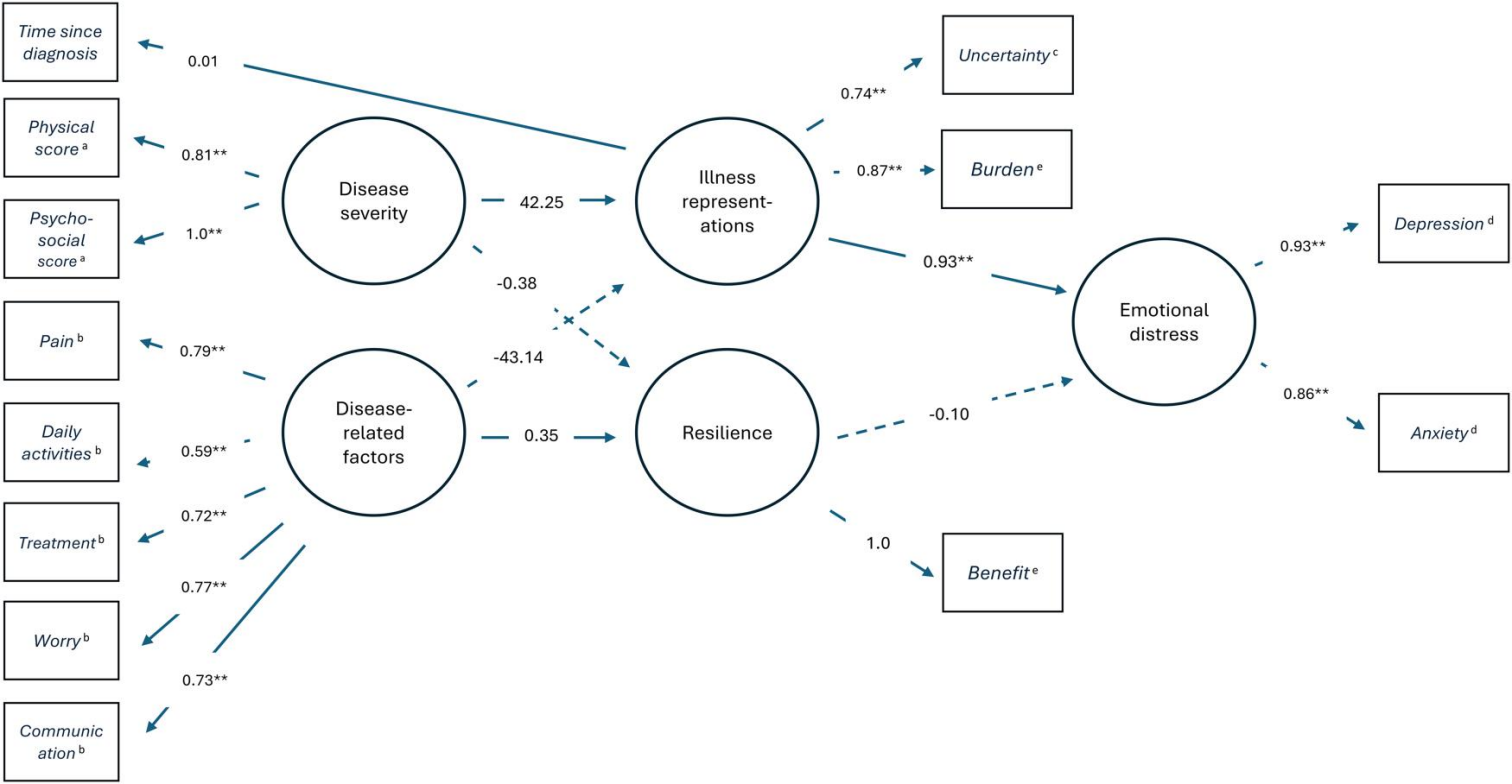
Estimated values of the hypothesised standard coefficients. Latent variables are represented by circles and observed variables by squares. The values represent standard estimates. Positive paths are represented by solid lines while negative paths are represented as broken lines. **P < 0.001. ^a^Subscores and total score as measured by the PedsQL 4.0 Generic Core Scale. ^b^Subscores as measured by the PedsQL 3.0 Rheumatology Module. ^c^Uncertainty as measured by the CUIS. ^d^Subscores and main score as measured by the PI-ED. ^e^Subscores as measured by the BBSC

Significant findings were observed for the proposed model (χ^2^ (52) = 109.8, *P* < 0.001). Overall, based on the criteria and cut-offs reported by Schermelleh-Engel *et al.* [43], the model was considered a good (GFI = 0.996, SRMR = 0.045, CFI = 0.949) to an adequate fit (RMSEA = 0.068, TLI = 0.924). Fig. 4 shows the estimated values of the hypothesised standardised path coefficients. Illness representations were found to impact on emotional distress (β = 0.93, *z* = 34.44, *P* < 0.001).

## Discussion

The aim of the current study was to test a new model of illness representations and resilience in relation to self-perceived anxiety and depression in CYP with JDM and to investigate overall HRQoL. Overall, the predicted model was found to be a good fit. It showed that illness representations impact on mental health, while resilience did not. However, as the χ^2^ test was significant, this suggests that the model is not a perfect fit and requires adapting, although having a significant result is common and often ignored when large sample sizes have been used [43].

Illness representations were measured to identify how CYP viewed their condition in relation to illness uncertainty and burden. Illness representations were the strongest predictor of emotional distress as assessed by self-reported anxiety and depression. This is supported by findings from other childhood chronic conditions such as cancer, asthma and type 1 diabetes that suggest self-perceived uncertainty impacts on emotional well-being [16–22]. Studies investigating the impact of illness burden in childhood chronic conditions have mostly focused on caregiver burden, while less is known in relation to self-perceived burden. Research suggests that caregiver burden is associated with parental depression and anxiety [45] as well as reduced QoL [46] and reduced caregiver capacity [25]. In teenagers with type 1 diabetes, perceived burden has been found to be associated with reduced physical and emotional health [47]. A qualitative study investigating the impact of disease burden in chronic diseases in adults identified factors that exacerbate perceived burden, including challenges with taking medications, confusion about medical information, role and activity limitations and psychosocial problems alongside other challenges [48]. In order to improve QoL in CYP with JDM, it is important to understand which factors amplify the disease burden. Similarly, a qualitative study addressing uncertainty in adults with chronic conditions found that clear communication with the healthcare team and trust improves coping with and managing uncertainty associated with chronic diseases [49]. More research needs to be conducted in CYP with JDM to understand the concepts of uncertainty and burden in this cohort to be able to recommend and develop suitable strategies to reduce perceived burden and uncertainty.

Benefit finding has previously been shown to mitigate emotional distress [27] and anxiety [28], suggesting that building resilience counteracts the negative effects associated with illness perceptions. However, these findings are not supported by the current model. Although a negative association between benefit finding and emotional distress was observed, this was small in magnitude and not significant. Future research should explore other avenues investigating how building resilience improves coping. A recent scoping review, addressing the concept of resilience in children with chronic conditions, suggested that resilience is a multidimensional concept that can be categorised into internal factors (such as cognitive, social and emotional competencies), disease-related factors and external factors [such as family, peer and contextual competencies (school, cultural and community-related factors)], which leads to complexities when analysing and comparing resilience outcomes [50]. Moreover, it suggests that personal traits and psychosocial functioning should not be ignored in the context of disease-related measures when addressing resilience to promote developing positive coping strategies in the light of adversity [50].

Our data are consistent with a model in which emotional distress is driven by perceived illness uncertainty and burden rather than disease severity and disease-related factors alone. Therefore, to improve mental health and well-being of CYP with JDM and their families, interventions should target CYP’s perceived burden and uncertainty directly. Other factors may influence illness representations instead. For example, caregiver-reported impairment as assessed via three domains of child functioning (mobility and self-care, physical activity and role activity in age-appropriate daily tasks) showed to be a better predictor of distress than severity of illness, irrespective of whether this was clinician assessed or caregiver reported [51].

This is the largest self-report HRQoL study that has been conducted with CYP with inflammatory myositis to date, and the first survey of HRQoL in JDM that has been completed by CYP in the UK. The data show that CYP with JDM experience impaired HRQoL compared with a healthy cohort and that poorer HRQoL and greater emotional distress were reported by those who perceived higher levels of unpredictability about their JDM and greater disease burden.

Compared with CYP with rheumatological conditions in a previous study [35], participants in the current study scored marginally lower on the treatment, worry and communication domains of the PedsQL, supporting previous results in those domains. However, the current cohort scored higher on the PedsQL pain and activities of daily living domains when compared with the overall rheumatology cohort, while they scored similarly to those of the small subsample of people with JDM in the Varni *et al.* study [35]. The lower mean scores in the overall rheumatology sample were attributed to CYP with fibromyalgia who scored lower on these domains than CYP with JDM, JIA and juvenile SLE.

The current study population also reported fewer emotional problems as measured by the PI-ED. However, the comparative cohort consisted of a clinical sample that included CYP from Child and Adolescent Mental Health Services and paediatric psychology services alongside paediatric outpatient departments rather than comparing results from a healthy cohort, and therefore no further comparisons can be made between those two cohorts.

There are several limitations that must be considered when interpreting the present results. First, the response rate of 50%, while high for questionnaire studies, opens the possibility that there was a bias in responders. One could speculate that those who were struggling with psychosocial difficulties might be more likely to respond, but also it could be that those with difficulties were less likely to respond. Also, children <8 years of age were not included in the sample, therefore conclusions should be applied to younger children with caution. Additionally, the current study only captured CYP self-report data. Future research could look into understanding the difference between self-report data compared with parent-proxy data [11, 12]. Discrepancies between self-report data have been well established, with some research suggesting that CYP have a tendency to report more symptoms than their parents and caregivers [11]. Less is known about the perceived impact on daily life relating to self-reported symptoms in CYP with JDM between CYP and their parents or caregivers and whether self-reported discrepancies between self-reported and parent-proxy data differ when there is active disease compared with when CYP are in remission. There may also be a proportion of CYP who are likely to underreport their symptoms to avoid further hospital visits, which can impact observed discrepancies between self- and proxy-reported data. Lastly, while the average age of diagnosis (7 years of age) and sex distribution (mostly girls) are indicative of the wider JDM picture across the UK and further afield, this study sample had not been seen in clinical care for an average of 1.8 years. Our knowledge of the current disease state and treatment is based on self-reported data. As a result, a future study should focus on testing our conceptual model by including clinical outcome data. This could be accomplished by recruiting CYP directly in clinics. The current study’s recruitment strategy focused on reaching out to all participants on the UK JDCBS registry, aiming to achieve widespread diversity through equality and inclusion. Therefore, a study testing the current conceptual model by including clinical data could further substantiate our findings.

## Conclusion

The aim of this study was to explore HRQoL and to test a new model of uncertainty and perceived burden in a self-reported UK sample of CYP diagnosed with JDM. Our model is consistent with the proposed model whereby illness representations, which were driven by uncertainty and burden, were identified as the strongest predictor of depression and anxiety. This highlights that there is a need to reduce uncertainty, which should be prioritised by clinical teams. Clear communication and increased knowledge could benefit those who struggle with the unpredictable disease course associated with JDM. To improve QoL and mental health, interventions should also aim to address resilience factors that mitigate against poor mental health outcomes and identify suitable strategies to increase coping in affected children and young people.

## Supplementary Material

rkag070_Supplementary_Data

## Data Availability

The data that support the findings of this study are available from the corresponding author upon reasonable request.
