# Supplementary material for: A test of a conceptual model of uncertainty, benefit and burden in children and young people with juvenile dermatomyositis
Source: Rheumatol Adv Pract. 2026 Jun 10;10(3):rkag070. doi: 10.1093/rap/rkag070 (PMC13335649; doi:10.1093/rap/rkag070)
Supplement: rkag070_Supplementary_Data [file rkag070_supplementary_data.docx]

**Supplementary Data S1. Clinical, research and administrative contributors to JDCBS members were as follows:**

Dr Kate Armon, and Ms Louise Coke, Ms Julie Cook, Ms Amy Nichols, Ms Vanja Briggs and Ms Emily Tropman (Norfolk and Norwich University Hospitals);Dr Liza McCann, Mr Ian Roberts, Dr Eileen Baildam, Ms Louise Hanna, Ms Olivia Lloyd, Susan Wadeson, Ms Michelle Andrews, Ms Olivia Lloyd, Mrs Jane Roach and Dr Beverley Almeida (The Royal Liverpool Children’s Hospital, Alder Hey, Liverpool); Dr Phil Riley, Ms Ann McGovern, Ms Verna Cuthbert and Ms Precious Iheke (Royal Manchester Children’s Hospital, Manchester); Dr Clive Ryder, Ms Janis Scott, Ms Beverley Thomas, Professor Taunton Southwood, Dr Eslam Al-Abadi and Ms Ruth Howman (Birmingham Children’s Hospital, Birmingham); Dr Sue Wyatt, Mrs Gillian Jackson, Dr Mark Wood, Dr Tania Amin, Dr Vanessa VanRooyen, Ms Deborah Burton, Ms Louise Turner, Ms Heather Rostron, and Ms Sarah Hanson (Leeds General Infirmary, Leeds); Dr Joyce Davidson, Dr Janet Gardner-Medwin, Dr Neil Martin, Ms Sue Ferguson, Ms Liz Waxman and Mr Michael Browne, Ms Roisin Boyle, Ms Emily Blyth, Ms Susanne Cathcart, Dr Kirsty McLellan and Dr Jaclyn Keightley (The Royal Hospital for Sick Children, Yorkhill, Glasgow); Dr Mark Friswell, Professor Helen Foster, Ms Alison Swift, Dr Sharmila Jandial, Ms Vicky Stevenson, Ms Debbie Wade, Dr Ethan Sen, Dr Eve Smith, Ms Lisa Qiao, Mr Stuart Watson and Ms Claire Duong, Dr Stephen Crulley, Mr Andrew Davies, Miss Caroline Miller, Ms Lynne Bell, Dr Flora McErlane, Dr Sunil Sampath, Dr Josh Bennet, Mrs Sharon King, Mr Christopher Long and Ms Lesley Brindley (Great North Children’s Hospital, Newcastle); Dr Helen Venning, Dr Rangaraj Satyapal, Mrs Elizabeth Stretton, Ms Mary Jordan, Dr Ellen Mosley, Ms Anna Frost, Ms Lindsay Crate, Dr Kishore Warrier, Ms Stefanie Stafford, Mrs Brogan Wrest, Ms Chia-Ping Chou, and Mr Paul Pryce (Queens Medical Centre, Nottingham); Professor Lucy Wedderburn, Dr Clarissa Pilkington, Dr Nathan Hasson, Dr Muthana Al-Obadi, Dr Giulia Varnier, Dr Sandrine Lacassagne, Ms Sue Maillard, Mrs Lauren Stone, Ms Elizabeth Halkon, Ms Virginia Brown, Ms Audrey Juggins, Dr Sally Smith, Ms Sian Lunt, Ms Elli Enayat, Ms Hemlata Varsani, Ms Laura Kassoumeri, Miss Laura Beard, Ms Katie Arnold, Mrs Yvonne Glackin, Ms Stephanie Simou, Dr Beverley Almeida, Dr Kiran Nistala, Dr Raquel Marques, Dr Claire Deakin, Dr Parichat Khaosut, Ms Stefanie Dowle, Dr Charalampia Papadopoulou, Dr Shireena Yasin, Dr Christina Boros, Dr Meredyth Wilkinson, Dr Chris Piper, Ms Cerise Johnson-Moore, Ms Lucy Marshall, Ms Kathryn O’Brien, Ms Emily Robinson, Mr Dominic Igbelina, Dr Polly Livermore, Dr Socrates Varakliotis, Ms Rosie Hamilton, Ms Lucy Nguyen, Mr Dario Cancemi, Dr Ovgu Kul Cinar, Dr Elena Moraitis, Ms Serena Cruickshank-Hull, Ms Klaudia Kupiec, Dr Hannah Peckham, Dr Qiong Wu, Dr Melissa Kartawinata, Ms Bethany Jebson, Ms Nia Evans, Mr Chadwick Pils, Ms Persephone Jenskins, Dr Afroditi Barmpakou, Dr Ali Mulla Issa, Dr Senne Cuyx, Dr Ryan Lethem, Dr Nagesha Muniyappa, Dr Zahoor Khan, Dr Mashal Shamsuddin, Dr Chenqu Suo, Dr Mariyah Albrahim, Dr Nkechi Maduaka, Dr Lilase Maduaka, Dr Samantha Cooray and Dr Rebecca Dancey (Great Ormond Street Hospital, London); Dr Kevin Murray (Princess Margaret Hospital, Perth, Western Australia); Dr Coziana Ciurtin, Dr John Ioannou, Mrs Caitlin Clifford, Ms Linda Suffield, Ms Maryam Butt, Ms Sydnee Pope and Ms Laura Hennelly (University College London Hospital, London); Ms Helen Lee, Ms Sam Leach, Ms Helen Smith, Dr Anne-Marie McMahon, Ms Heather Chisem, Ms Jeanette Hall and Ms Amy Huffenberger (Sheffield’s Children’s Hospital, Sheffield); Dr Nick Wilkinson, Ms Emma Inness, Ms Eunice Kendall, Mr David Mayers, Ms Ruth Etherton, Ms Danielle Miller and Dr Kathryn Bailey (Oxford University Hospitals, Oxford); Dr Jacqui Clinch, Ms Natalie Fineman, Ms Helen Pluess-Hall, Ms Suzanne Sketchley, Ms Melanie Marsh, Ms Anna Fry, Ms Maisy Dawkins-Lloyd, Ms Mashal Asif, Ms Gigi Leung and Mr Alexander Smith (Bristol Royal Hospital for Children, Bristol); Dr Joyce Davidson, Margaret Connon and Ms Lindsay Vallance (Royal Aberdeen Children’s Hospital); Dr Kirsty Haslam, Ms Charlene Bass-Woodcock, Ms Trudy Booth, and Ms Louise Akeroyd (Bradford Teaching Hospitals); Dr Alice Leahy, Amy Collier, Rebecca Cutts, Emma Macleod, Dr Hans De Graaf, Dr Brian Davidson, Sarah Hartfree, Ms Elizabeth Fofana and Ms Lorena Caruana (University Hospital Southampton); Dr Catriona Anderson (Royal Hospital for Children and Young People, Edinburgh); Dr Jayne MacMahon and Dr Peter Bale (Cambridge University Hospitals).
